# Characterization and antioxidant effect of mucilage in leaves from *Cocculus hirsutus*

**DOI:** 10.6026/973206300200439

**Published:** 2024-05-31

**Authors:** Devasahayam Leema Rose Mary, Antonysamy Lawrance, Arokiadoss Edwina Sherley Felicita, Pushpam Marie Arockianathan

**Affiliations:** 1 PG & Research Department of Biochemistry, St. Joseph's College of Arts & Science (Autonomous), Cuddalore - 607001, Tamil Nadu, India

**Keywords:** *Cocculus hirsutus*, Mucilage, Antioxidant, Cytotoxicity

## Abstract

Natural polysaccharides are attractive biodegradable polymers. Among the natural plant-based polysaccharides, mucilage is an interest for numerous biomedical applications. Hence, mucilage was isolated from the leaves of *Cocculus hirsutus* (Family; *Menispermaceae*) and tested for its phytochemicals, physio-chemical characteristics using standard procedure such as solubility, pH, swelling index *etc.,* and structural characterization studies using FTIR, GC-MS and SEM followed by antioxidant and *in vitro* cytotoxic assays. The phytochemical results showed the presence of carbohydrates, amino acids, flavonoids, alkaloids, tannins, terpenes, saponin, glycosides and steroids. The yield percentage of mucilage was 26% and showed swelling index of 6.8-7.4. The FTIR spectra of mucilage showed characteristics strong peaks of major functional groups. The SEM image showed the porous and rough surface morphological characters of mucilage. The obtained mucilage showed antioxidant potential by DPPH, FRAP and Total reducing power assay and also exhibited non-cytotoxic character against fibroblast cell lines. Thus, the isolated mucilage showed promising characteristics which can be exploited for various biological applications from food to drug release studies.

## Background:

Nature has provided many natural polymers with an ability to withstand chemical modifications to suit for wide range of applications; it can be exploited to its best since it's readily available and has good potential to degrade. It possesses various characteristics like non-toxic, good mechanical strength, inexpensive, inert to host tissue and biocompatible that makes them an efficient choice for its versatile applications. Due to their hydrophilic and cost-effective nature, it was given positive regulatory acceptance for large scale production [1, 2]. Different polymers like chitosan, cellulose, alginate, gum, mucilage *etc.,* were found abundantly in nature and they were obtained from different sources. The polymers like chitosan and alginate from marine sources, cellulose, gum, mucilage *etc.,* from plant sources and collagen, gelatin *etc.,* from animal sources which were exploited for various biological applications. These polymers constitute structurally wide class of biological substances with broad array of physicochemical inherent properties like coating agent, gel forming, controlled release matrix, mucoadhesive and permeation property. These properties help them to blend with active compound to form various drug formulations by acting as protective agent and to enhance the bioavailability or the effectiveness of the drug in the formulation during storage and use. Many studies have been carried out in the fields of food technology and pharmaceuticals using these polymers [3, 4]. Among the different polymers, plant-based polymers showed considerable functional properties which can be used in several industries. Among the plant polysaccharide, mucilage showed promising scope in research due to their specific functionalities. Mucilage was a complex polymer, capable of becoming viscous in the presence of water. [5] Mucilage was obtained from different parts of plants like fruits, leaves, seeds and other sources [6, 7]. It has wide range of advantages because of its biodegradability, biocompatibility, low cost, eco-friendly, easily available and abundance in nature. It was also preferred for synthetic and semi synthetic excipients due to their non-irritating, emollient and non-toxic nature. The mucilage obtained from plants exhibit characteristic properties like binder, thickeners, emulsifiers, stabilizer, dissolving, suspending, film forming and gelling agents and which can be exploited for several industrial applications [8, 9]. Among the industries, pharma sector used mucilage for their diverse applications like demulcent properties, dental adhesives, bulk *laxative*s, tablet binder, film forming agents, buccal tablets *etc.* It also been used as matrices for controlled and prolonged release of drugs. Mucilage consists of different monosaccharides that mostly combine with uronic acids in different proportions. Upon hydrolysis, they yield glucose, galactose, mannose, arabinose, xylose and organic acids [10, 11]. The presence of sugars in mucilage of *Urginea indica* was studied using HPLC analysis. It was reported in the previous studies that many plant mucilage was studied and characterized such as *Aloe vera*, [12] *Trigonella foenum graecum* (fenugreek), [13] *Mimosa pudica*, [14] *Opuntia ficus-indica L*.,[15] *Lepidium sativum*, [16] *Ocimum basilicum LM.S*., [17] and *Hibiscus rosa-sinensis* [18].

*Cocculus hirsutus* plant belongs to the family of *Menispermaceae*. It was extensively spread throughout tropical and subtropical regions, including Sudan, China, India, and central Asia. It contains polysaccharides and a gelatinous type of materials; it was not absorbed in the G.I.T and also non- toxic to human skin. The presence of alkaloids, flavonoids, fixed oils, fats, glycosides and phytosterol was found in the leaves of *Cocculus hirsutus*. Numerous bioactive compounds, including 2,3,4,5-Tetra hydroxy pentanal, Vitamin E, quinic acid, linolenic acid, phthalic acid, stearic acid, beta-sitosterol, camp sterol, lupeol, squalene and betulin have been found in the GC-MS results. These compounds have a wide range of established therapeutic uses [19]. *Cocculus hirsutus* was used traditionally in various ailments like *diuretic*, *laxative*, [20] analgesic and anti-inflammatory activities, wound healing activity, hepatoprotectives, anti-diabetic activity [21] and anticancer activity [22]. It has some active constituents like flavonoids, triterpenoids and saponins in the ethanolic extract of *Cocculus hirsutus* leaves showed anti-arthritic activity in dose-dependent manner. This extract also showed significant antioxidant activity due to the presence of flavonoids, antioxidant, vitamins and phenolic compounds [23]. It was also used in the treatment of ulcers, Tuberculosis and spermatogenic activity. The leaves were also used as an aphrodisiac, to treat gonorrhea, eczema, dysuria, polyuria, rheumatoid arthritis, fevers, piles, syphilis, blood disorders, cough, ophthalmia, cephalalgia and neuralgia [24]. *Cocculus hirsutus* showed high percentage of mucilage but its applicability was very little exploited, and it was used only as a gelling agent for Flurbiprofen drug [25]. Moreover, this mucilage was not characterised earlier. Hence, the mucilage was isolated from the leaves of Cocculus hirsuus and it was characterized for various physicochemical properties. The extracted mucilage was also tested for its antioxidant potential and cytotoxicity using fibroblast cell lines.

## Materials and Methods:

## Collection and authentication of Plant:

The plant *Cocculus hirsutus* was collected from Cuddalore district, Tamil Nadu, India. This plant was identified by Rapinat Herbarium, (No; B.A.001/3.1.2020) St. Joseph's College, Tiruchirappalli, India. The leaves were separated and then washed with pure water; shade dried for 2 to 3 weeks. After that the dried leaves were reduced to a fine powder, and then stored in an airtight container for further use.

## Extraction of Mucilage from *Cocculus hirsutus* leaves:

The dried leaves powder was soaked in water for 1 hour, boiled for 30 minutes and kept aside for complete release of the mucilage into the water and filtered through eight-fold muslin cloth. An equal volume of ethanol was added to the filtrate for subsequent precipitation. The precipitated mucilage was dried in an oven and then grounded into fine powder using a mortar and pestle. Finally, it was weighed and stored in desiccators.

## Physicochemical Characterization of Isolated Mucilage:

The powdered mucilage of *Cocculus hirsutus* were applied to different physicochemical test. The organoleptic properties like colour, odour and texture, physical properties like solubility, pH, moisture content, Ash content, swelling index, emulsifying capacity and viscosity and the phytochemicals screening, antioxidant and cytotoxic activity of the mucilage was studied.

## Quantification of Mucilage:

The percentage yield of obtained mucilage was determined by dividing the weight of dried mucilage obtained by the weight of dried leaf powder:

Percentage yield = Weight of dried mucilage obtained/ Weight of leaves power used x 100.

## Phytochemical screening of plant mucilage:

Various identification tests were performed on the extracted mucilage to conduct phytochemical investigations. The following tests were run: the Molisch test for carbohydrates; the Fehling and Benedict tests for reducing sugars; the Iodine test for starch; the Ninhydrin and Millon tests for proteins and amino acids. Tannins (Ferric chloride, lead acetate test), Glycosides (Legal, Bontrager's test), alkaloids (Mayer, Dragendroff's test), and steroids (Liebermann-Burchard's test).

## Characterisation of Isolated mucilage:

The structural characteristics were analysed using FTIR, GC-MS and SEM. The mucilage was also tested for its antioxidant activity and cytotoxic activity.

## Fourier Transform Infrared(FT-IR)Spectral Studies:

The rapid and affordable method of determining a compound's functional group is the FTIR spectrum. The fine KBr powder (200 mg) was combined with about 1% dried mucilage, and then the mixture was finely ground and placed into a pellet-forming die (KBr press, Karnavati, India). Transparent pellets are the result of applying a force of about eight tons for five minutes while under vacuum. The pellet was placed onto an infrared spectroscopy holder for Fourier transform measurements. The wave number range of 4000 to 400 cm-1 was utilized to record the spectrum in order to determine the structural characteristics and functional groups of the mucilage sample.

## GC-MS analysis:

Bioactive compounds in the mucilage identified by Gas chromatography and Mass spectroscopy (GC-MS). This analysis was done by standard protocol using Agilent technologies GC systems with GC-7890B/MS-7000D model (Agilent Technologies, Santa Clara, CA, USA) equipped with HP-IMS column (60 m in length x 0.250 mm in diameterx1.0 µm in thickness of film). Spectroscopy detection by GC-MS involved an electron ionization system which utilized high energy electrons (70 eV). Pure helium gas (99.995%) was used as the carrier gas with flow rate of 1 ml/min. The temperature of injector was 3000C and the sample was injected in the split mode with a split ratio 10:1. Helium (He) was used as carrier gas, and the flow rate of gas 1.0 ml/min. The temperature of MSD transfer line was at 2800C.For mass spectra determination MSD was operated in electron ionization (EI) mode, with the ionization energy of 70 eV, while the mass range scanned was 50-550 m/z. The temperature of ion source was 3000C and that of MS quadruple was 1500C. The identification of components was based on comparison of their mass spectra with those of NIST mass spectral library.

## SEM:

The mucilage samples have been attached to an aluminium stub using double-sided tape. First, the tape was securely fastened to the stub, and then the powder sample was gently distributed across its surface. To make the sample conductive, a thin layer of gold was applied to the stub containing the sample. Using Scanning Electron Microscopy (Philips, Lancashire, XL 30), the photomicrographic pictures of the samples were acquired.

## *in vitro* Antioxidant activity:

## DPPH assay:

Mucilage's capacity to scavenge DPPH radicals was evaluated utilizing the Blois *et al* method [26]. 2.5 ml of a 0.5 mM DPPH solution in methanol was combined with an aliquot of 0.5 mL of the sample solution in methanol. After giving the mixture a vigorous shake, it was allowed to stand at room temperature for 30 minutes in the dark. At 517 nm, the absorbance was measured in relation to the blank. A positive control was ascorbic acid. The formula used to determine the percent (%) of inhibition of the DPPH free radical was:

% inhibition = absorbance of control - absorbance of sample / absorbance of control x 100.

## FRAP assay:

Ferric reducing power of mucilage were determined using FRAP assay. [27, 28] This method is based on the reduction of colourless ferric complex (Fe3+ tripyridyl triazine) to blue-coloured ferrous complex (Fe2+ tripyridyl triazine) by the action of electron donating antioxidants at low PH. The working FRAP reagent was prepared by mixing 10 volumes of 300mM acetate buffer, pH 3.6, with 1 volume of 10mM TPTZ (2,4,6-tri(2-pyridyl)-triazine) in 40mM HCl and with 1 volume of 20mM ferric chloride. All the required solutions were freshly prepared before their uses. 100 µL of samples (mg/mL) were added to 3mL of prepared FRAP reagent. The reaction mixture was incubated in a water bath for 30min at 37°C. Then, the absorbance of the samples was measured at 593 nm.

## Total Reducing Power assay:

The total reducing power was determined by the Oyaizu *et al* method.[29] Different concentrations of test material in 1 ml 200 µM potassium phosphate buffer, pH 6.6, and 2.5 ml 1% potassium ferricyanide [K_3_Fe (CN)_6_]. The mixture was incubated at 50°C for 20 min. A 2.5-ml aliquot of 10% trichloroacetic acid was added to the mixture, which was then centrifuged at 3000 g for 10 min. The upper layer of the solution (2.5 ml) was mixed with 2.5 ml distilled water and 0.5 ml 0.1% FeCl3and absorbance was measured at 700 nm. Butylated hydroxytoluene (BHT) 5, 10, 20, 40, 50 µg was used as a reference material. Higher value absorbance of the reaction mixture indicated greater reducing power. All tests were performed in triplicate and the graph was plotted with the average of the three determinations.

## Cell viability assay:

The Fibroblast cell lines (L929) were procured from National Centre for Cell Science, Pune, India. Separate 96-well plates were used to plate the fibroblast cell lines (L929) at a concentration of 1x10^4^ cells/well in EMEM media containing 10% fetal bovine serum (HI media, India) and 1X antibiotic antimycotic solution. The plates were kept in a CO_2_ incubator at 37°C with 5% CO_2_. The cells underwent a 200 µL 1X PBS wash before being treated with different concentrations of the sample in serum-free media and incubated for a full day. At the end of the treatment period, the medium was aspirated from the cells. After adding 0.5 mg/mL MTT prepared in 1X PBS, the mixture was incubated for 4 hours at 37°C in a CO2 incubator. Following the incubation period, 200 µL of PBS was used to wash the medium containing MTT out of the cells. After dissolving the crystals with 100 µL of DMSO and mixed thoroughly. At 570 nm, a change of colour intensity was assessed. The formazan dye changes to a blue-purple colour. The microplate reader was utilized to measure the absorbance at 570 nm.

## Results and Discussion:

## Phytochemical screening:

The powdered mucilage was analyzed for its phytochemicals; it showed the presence of carbohydrates, aminoacids, flavonoids, tannins, alkaloids, saponins and glycosides (Table 1). Many mucilage and gums have significant anticoagulant, hypoglycaemic, anticancer, anti-inflammatory, and wound healing properties which make them very exciting constituents for many biological applications. With increasing demand and resurgence of traditional medicines, these plant products created interest in the minds of researchers to produce safe and efficient drug formulation for the consumers.

## Physicochemical characterization:

## Organoleptic Properties:

The isolated mucilage of *Cocculus hirsutus* has important industrial uses in the pharmaceutical and food sectors due to its physicochemical characteristics. The isolated mucilage organoleptic characterization revealed a distinct smell and an amorphous, light green powder. It produced a neutral, greenish solution that was slimy and colloidal when dissolved in water. Molisch's test verified that the mucilage contained carbohydrates. A yield of 26% mucilage was obtained from the leaves of *Cocculus hirsutus*.

It was discovered that the mucilage swelled in cold water and disintegrated when shaken vigorously. While it was easily dissolved in hot water, it was discovered to be insoluble in acetone, chloroform, and ethanol. 6% of the mucilage weight was lost during the drying process, but it seems that some moisture was still present and could aid in interactions with other materials. It was found that the pH of a 1% mucilage solution was 6.8. This means that when mucilage was consumed orally, it causes less irritation to the gastrointestinal tract and mucous membranes.

## Swelling capacity:

Mucilage's increased ability to swell was accompanied by increased water-holding capacities [30]. Increased swelling resulted in increased surface area, surface wet ability, and water penetration, which forms more hydrophilic biofilm matrices that may be readily biodegradable. *C.hirsutus* mucilage was found to have a swelling index of 6.9-7.2. FTIR data suggested that the presence of hydrophilic groups, like hydroxyl groups, might be the cause of this.

## Emulsifying capacity:

Emulsifying capacity (EC) was responsible for the stabilization and formation of emulsion. The emulsifying capacity of *Cocculus hirsutus* mucilage showed higher percentage when compared with okra mucilage (52 %), *Ocimum canum* seed mucilage (74.41%) and Locust bee gum (52%) [31]. The mucilage from different plants was mixed with other constituents to be used in food packaging industries. This was mainly due to the presence of emulsification properties of polysaccharide to form homogenous solution either blended with other substances or singly. This property was utilized to make a low-cost, compatible, antimicrobial material with desired tailor-made properties as reported previously [32].

## Viscosity:

Studies on the viscosity of a 1.0% w/v solution of isolated mucilage at various temperatures revealed that viscosity increased as temperature increased. Thus, it was discovered that the polysaccharide-rich mucilage had superior physicochemical characteristics, including a neutral pH, a higher swelling index, and good flow characteristics.

## Fourier Transform Infrared(FT-IR)Spectral Studies:

**Figure 1:**
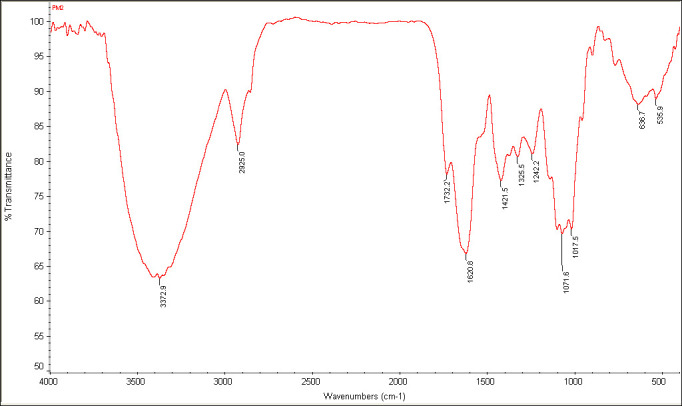
FTIR spectrum of *Cocculus hirsutus* leaves mucilage.

The molecular and material structure of the polymer has been extensively characterized through using the technique of FTIR spectroscopy. Functional groups and the ways in which they cling to the polymer backbone are frequently identified through FTIR spectroscopy characterization [33]. The FTIR spectra of *Cocculus hirsutus* mucilage showed typical bands and peak characteristic of mucilage in Figure 1. A broad peak around 3372.9 cm^-1^ indicates the presence of OH group in sugars which was the main component of mucilage. The functional OH group in the mucilage can bind with water molecules and can swell. It also gives hydrophilic character for mucilage. The peak at 2925 cm^-1^, mainly attributes to symmetric and asymmetric vibrations of the C-H grouping of methyl and methylene from monosaccharide units. [34] The peak at 1732.2 cm^-1^ was mainly due to C=O stretch in aldehyde group and a strong peak at 1620.8cm^-1^ was observed due to the presence of amide I deformation/C=O asymmetric stretching. The presence of C-O-C ether group was observed at 1017.5cm^-1^.

## GC-MS analysis:

**Figure 2:**
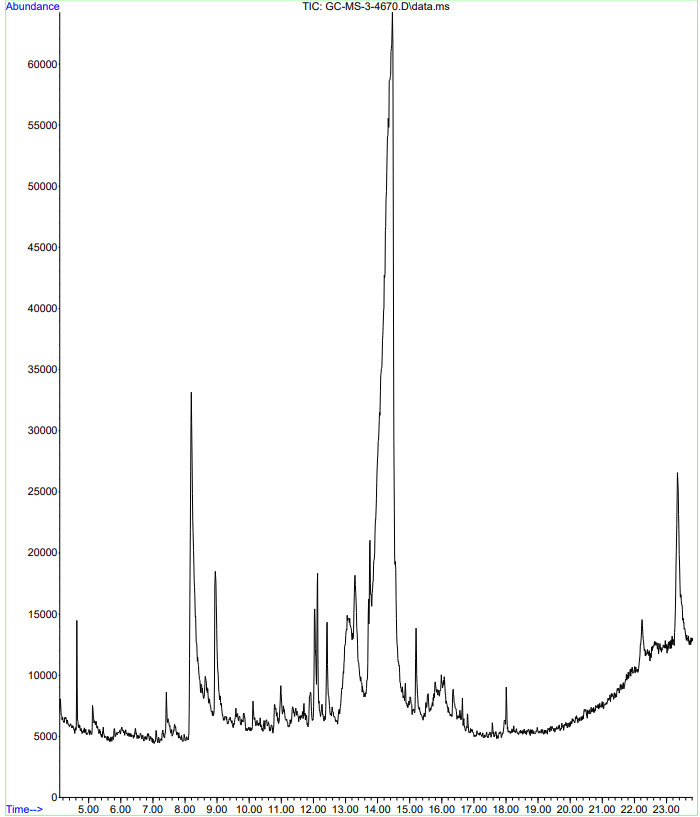
GC-MS chromatogram of *Cocculus hirsutus* mucilage

In the GC-MS chromatogram of *C. hirustus* mucilage (Figure 2) there were 30 peaks corresponds to 30 compounds present in it. Among them, only 3 compounds were in major composition in the analysed sample as identified by its Gas chromatogram. In the GC chromatogram, the first major compound was observed at a retention time of 14.463 min with area percentage of 57.35%. The second major compound was observed at a retention time of 8.197 min with area percentage of 10.91%. The third major compound was observed at a retention time of 23.340 min with area percentage of 4.62% (Table 3). From the library search report of the Mass spectra of these compounds, it was observed that the major compounds present in the analysed sample were alpha-d-Riboside, 1-O-dodecyl-, Methyl-d-glycero-beta-d-gulo-heptoside and Allo-Inositol. Also, it was evident from the literatures that, these compounds which belongs to the group of sugar series, present in these Cocculus family mucilage. Hence, it was concluded that, alpha-d-Riboside, 1-O-dodecyl-, Methyl d-glycero-beta-d-gulo-heptoside and Allo-Inositol were the major compounds present in the analysed sample. The other compounds present in minor amounts were DL-Arabinose, Inositol,1-deoxy-, beta-D-Glucopyranose, D-Galactose,3-Aminopiperidin-2-one, n-Decanoic acid, Heptanoic acid, 1,5-Anhydro-l-rhamnitol, beta.-D-galactopyranosyl- and D-Allose.

## Scanning electron microscopy:

**Figure 3:**
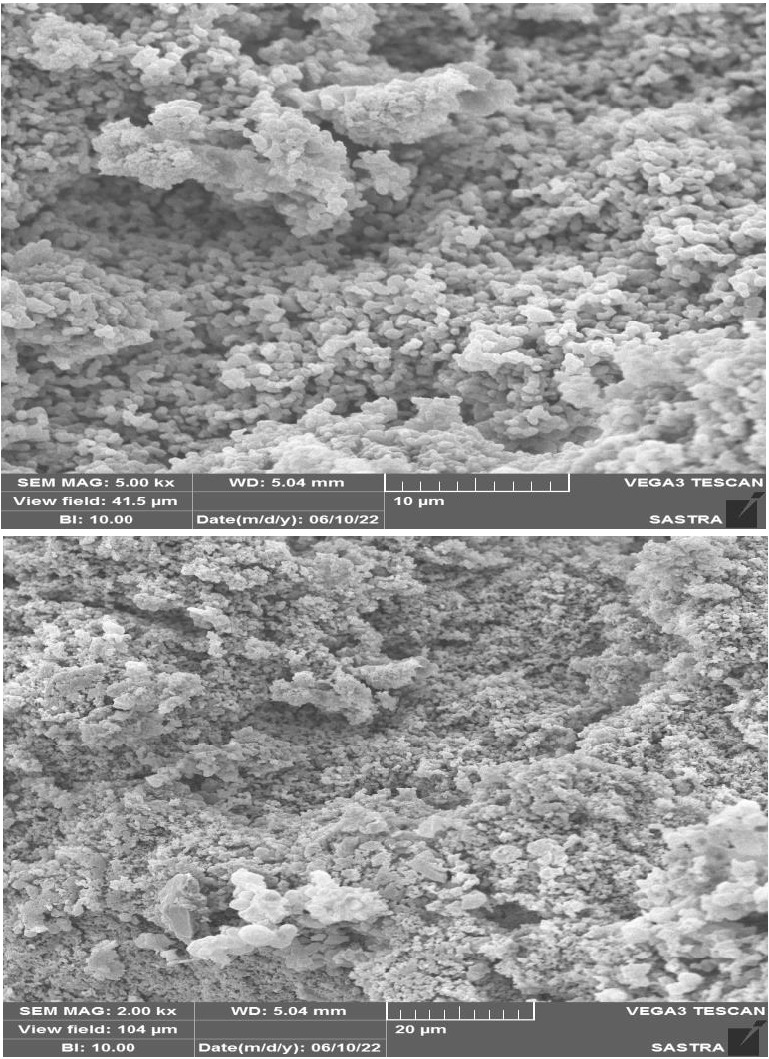
SEM image of dried *Cocculus hirsutus* leaves mucilage at different magnification.

Figure 3 showed various magnifications of the mucilage obtained from scanning electron microphotographs (SEM). The mucilage's amorphous nature was shown in the microphotographs. Most of the particles were observed as fibrous aggregates with erratic sizes and shapes. The SEM results implied that the ability of the mucilage to hold water depends on its surface characteristics. The process used to extract and purify the product may have an impact on the mucilage's structure, form, and surface topography. [35] Gums' hydration behaviour was influenced by their specific surface area and particle size, which also affects their intrinsic viscosity and molecular mass [36].

## Antioxidant activity:

## DPPH Assay:

**Figure 4:**
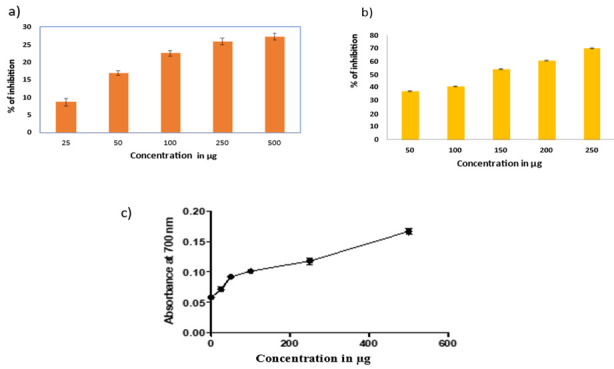
Antioxidant activity a) DPPH Assay b) FRAP assay and c) Total reducing power assay.

Plant polysaccharides like mucilage provide good antioxidant potentials due to the presence of reducing sugars. [37] This antioxidant property exhibits protective action against various diseases. The DPPH scavenging assay is most commonly used method to evaluate antioxidant potentials. *in vitro* antioxidant activity of isolated mucilage was increased with increase in concentration, and it shows maximum inhibition at 500µg (Figure 4a). The results indicated that the isolated mucilage from *C. hirsutus* leaves can effectively exhibiting its antioxidant potential. This phenomenon of antioxidant potential of different plant mucilage was already reported in the previous studies. [38- 39]

## FRAP Assay:

The antioxidant activity of any plant material can't be authenticated or established by a single antioxidant test. The different methods were used to assess the antioxidant potential of the plant material. [40] The present studies FRAP assay was used to assess the ability of the mucilage to reduce the ferric ion to ferrous. The formation of ferrous ion can be assessed by absorbance at 700nm [41]. The mucilage showed increased absorbance value at this wavelength which predicts its increasing reducing power. The results demonstrated that an increase in the concentration of mucilage showed increase in the reducing power (Figure 4b). The presence of various phytochemicals in the mucilage was responsible for the antioxidant activity which correlates with our qualitative phytochemical analysis.

## Total reducing power assay:

The reducing power of the mucilage was assessed by the principle of reduction of Fe^3+^ to Fe^2+^ which reacts with ferric chloride to form ferric ferrous complex. The reducing power in this assay was evaluated by the ability to donate an electron by the plant material or antioxidants [42]. The antioxidant present in the mucilage will reduce Fe^3+^ to Fe^2+^ by producing different colour shades depending on its reducing potential. [43] The higher reducing power of the mucilage was shown by higher absorbance value. The reference standard BHT showed higher reducing power than mucilage concentration. The reducing power of the mucilage increased with increase in concentration (Figure 4c). The different assays proved the antioxidant potential of the mucilage even though its reducing potential was lower than their reference standard.

## Cell viability assay:

**Figure 5:**
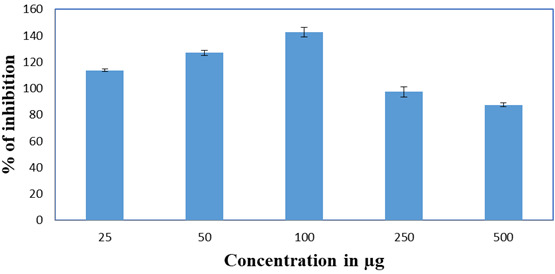
Cytotoxic activity of *Cocculus hirsutus* leaves mucilage.

**Figure 6:**
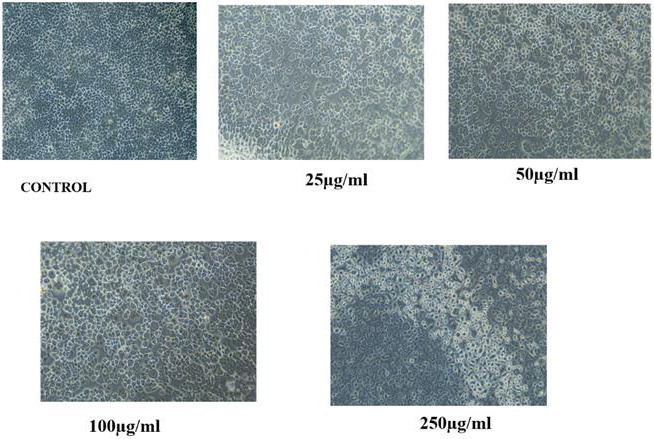
Cell viability images of fibroblast cell lines (L929) in different concentration of *C. hirsutus mucilage.*

The mucilage extracted from *Cocculus hirsutus* was screened for its cytotoxicity against normal fibroblast cell lines (L929) at different concentration by MTT assay. The result obtained was depicted in the Figure 4. Compared to the control group, the increase in the concentration of mucilage showed increase in the proliferation of cell lines. The increase in concentration greater than 100µg/mL showed less proliferic effect (Figure 5 and Figure 6). This clearly indicates that the mucilage of *Cocculus hirsutus* leaves did not show any cytotoxic effect on fibroblast cell lines, so it can be blended with the other natural substance to form biocompatible products.

## Conclusion:

Mucilage contains more phytochemicals with good functional properties like antioxidant activity and cytotoxic activity. Thus, the characteristics and functional properties of *Cocculus hirsutus* mucilage must be further explored mainly as potential additives/excipients for various industrial products in food and pharma sectors.

## Ethical approval:

The conducted research is not related to either human or animal use.

## Declaration of competing interest:

The authors declare that they have no known competing financial interests or personal relationships that could have appeared to influence the work reported in this paper.

## Declaration of generative AI and AI-assisted technologies in the writing process:

During the preparation of this work the author(s) not used AI assisted technologies in writing this manuscript.

## Figures and Tables

**Table 1:** Qualitative analysis of Carbohydrates, Amino acids and Phytochemicals

**S.No**	**Test name**	***Cocculus hirsutus*** **mucilage**
**A**	**Carbohydrates**	
a)	Molisch's test	++
b)	Fehling's test	++
c)	Benedict's test	++
d)	Barfoed's test	++
**B**	**Amino acids**	
a)	Ninhydrin test	++
b)	Millon's test	++
**C**	**Phytochemicals**	
a)	Alkaloids	++
b)	Flavonoids	++
c)	Tannins	++
d)	Saponins	++
e)	Glycosides	++
f)	Terpenes	++
g)	Steroids	++
The results were expressed as
(+) for the presence and
(-) for the absence of phytochemicals.

**Table 2:** Physicochemical characterization of *Cocculus hirsutus* leaves mucilage

**S. No**	**Parameters**	***Cocculus hirsutus*** **mucilage**
1	Solubility	
	Cold Water	Swell to form a gel
	Hot Water	Soluble
	Ethanol	Insoluble
	Acetone	Insoluble
	Chloroform	Insoluble
2	% of yield	26%
3	Swelling index	17
4	Moisture content	6%
5	Ash content	6.10%
6	pH	6.8
7	Swelling capacity	6.9-7.2
8	Emulsifying capacity	82%
9	Protein content (Lowry's method)	3.5mg/100 mL
10	Carbohydrates (OT method)	4.7mg/100 mL

**Table 3:** Major components present in the sample as per GC-MS analysis

**S. No**	**Retention Time**	**Percentage Composition (Area %)**	**Name of the Compound**
1	14.463	57.35	alpha-d-Riboside, 1-O-dodecyl-Methyl d-glycero-beta-d-gulo-heptoside Allo-Inositol
2	8.197	10.91	Benzoic acid, silver (1+) salt
3	23.34	4.62	2-Ethylacridine 1,2-Bis(trimethylsilyl)benzene

## References

[1] Ameri A (2015). Pharm. Biol..

[2] Carina Faccio AF (2015). Carbohydr. Polym..

[3] Gulden G (2023). Int. J. Biol. Macromol..

[4] Archana G (2013). Carbohydr. Polym..

[5] Zhao QS (2009). Carbohydr. Polym..

[6] Mir Hossein. H (2012). Food Res. Int.

[7] Akbari I (2014). J. Superscript. Fluids..

[8] Kuldeep S (2009). AAPS Pharm Sci Tech..

[9] Prajapati VD (2013). Carbohydr. Polym..

[10] Cai M (2013). Environ. Sci. Pollut. Res..

[11] Mariel M (2017). Journal of Chemistry..

[12] JG de Oliveira F (2021). Food hydrocolloids for Health..

[13] Mishra A (2006). Carbohydr. Polym..

[14] Ahuja M (2013). Int. J. Biol. Macromol..

[15] Maria Carolina Otalora (2021). polymers..

[16] Marva Ali R (2021). Int. J. Foods..

[17] Hosseini MS (2020). Int. J. Biol. Macromol..

[18] Ameena K (2010). Asian Pacific Journal of Tropical Medicine..

[19] Meena MK (2014). Int. J. Pharm. Pharm. Sci..

[20] Ganapaty S (2002). Fitoterapia..

[21] Ranjan PB (1993). Int. J. Pharm. Sci. Nano technol..

[22] Thavamani BS (2014). Pharm. Biol.

[23] Rakkimuthu R (2012). Int. J. Phytomedicine..

[24] Rajan Logesh (2020). Medicines..

[25] Mallikarjuna Rao K (2010). Int. J. Pharm Tech Res..

[26] Blois M (1958). Nature.

[27] Dudonne S (2009). J. Agricultural & Food Chemistry..

[28] Luqman S (2012). Evidence-Based Complementary and Alternative Medicine..

[29] Makoto Oyaizu M (1986). Japan Journal of Nutrition..

[30] Sikareepaisan P (2011). Carbohydr. Polym..

[31] Alpizar-Reyes E (2017). J. Food Eng..

[32] Maciel JS (2008). Carbohydr. Polym..

[33] Hindustan Abdul Ahad J (2011). greenpharmacy.info..

[34] Tavares SA (2011). Ciênciae Agrotecnologia..

[35] Qian JY (2009). Carbohydr. Polym..

[36] Wang Q (2006). Carbohydr. Polym..

[37] Kardosova A (2006). Fitoterapia,.

[38] Motiwala MN (2015). Bio act. Carbohydr. Diet. Fibre..

[39] Bayar N (2016). Int. J. Biol. Macro mol..

[40] Akinmoladun AC (2010). J. Med. Food..

[41] Mac Donald-Wicks LK (2006). J. Sci. Food Agric..

[42] Yildirim A (2000). J. Agric. Food Chem..

[43] Ferreira ICFR (2007). Food Chemistry..

